# Augmenting laboratory COVID serology data granularity for SARS-CoV-2 reporting.

**DOI:** 10.23889/ijpds.v7i3.1887

**Published:** 2022-08-25

**Authors:** Esmond Urwin, Andy Harris, Jenny Johnstone, Erum Masood, Antony Chuter, Michael Ferguson, Joanne Martin, Neil Sebire, Philip Quinlan, Emily Jefferson

**Affiliations:** 1 University of Nottingham; 2 National Pathology Exchange; 3 University of Dundee; 4 Pain UK; 5 Queen Mary University of London; 6 UCL Great Ormond Street Hospital Institute of Child Health

## Objectives

The global COVID pandemic has highlighted the need for the reporting of enhanced serology data from laboratory testing data. Qualitative results alone do not provide detailed data to understand levels of SARS-CoV-2 seroprevalence within a populace. To enable this, a minimum data set is required to support standardised data reporting.

## Approach

The CO-CONNECT project is concerned with the response to COVID within the UK and how datasets can be made Findable, Accessible, Interoperable and Reusable (FAIR). One aspect of this is a focus upon improving granular levels of COVID serology testing data being reported by national laboratories to the National Pathology Exchange (NPEx). A study was conducted to ascertain the essential data elements that could formulate a COVID Serology Data Standard (CSDS). Three NHS laboratories acting as pilot studies and NPEx have been actively involved with the development of the CSDS, together with clinicians and academics from the CO-CONNECT project.

## Results

Feedback from clinicians, academics and NPEx has been collected, together with NHS laboratory feasibility analysis to derive a minimum set of data elements that form a proposed data standard to standardise the reporting of laboratory COVID serology data for the UK. The proposed CSDS comprises twelve data elements in total, grouped into four main areas, (1) subject identifier and test date and time, (2) analyser types, test kits and samples, (3) qualitative results and (4) quantitative results. To further support the standardisation effort, a proposed Health Level Seven (HL7) message structure has been created to enable the reporting of the CSDS data elements to NPEx.

## Conclusion and Relevance

The CSDS has been created to help augment levels of reported granular data for laboratory COVID serology testing. If adopted, this could enable healthcare professionals to better understand the calibration of assays across multiple analyser types and thus antibody response levels.

